# Copper biosorption by *Serratia plymuthica*: crucial role of tightly bound extracellular polymeric substances in planktonic and biofilm systems

**DOI:** 10.1007/s10532-026-10245-6

**Published:** 2026-01-16

**Authors:** Alice Melzi, Sarah Zecchin, Milena Colombo, Gigliola Borgonovo, Stefania Mazzini, Subhoshmita Mondal, Stefania Arioli, Lucia Cavalca

**Affiliations:** Dipartimento di Scienze per gli Alimenti, la Nutrizione e l’Ambiente (DeFENS), Università degli Studi di Milano, Via Celoria 2, 20133 Milan, Italy

**Keywords:** *Serratia plymuthica*, Extracellular polymeric substances, Biofilm, Biosorption, Copper, Electroplating wastewater

## Abstract

Heavy metals in aquatic environments pose significant environmental and human health risks, highlighting the urgent need for innovative remediation strategies. This study explores the role of bacterial extracellular polymeric substances as active binding surfaces for copper, in planktonic cells and biofilm-based adsorption systems. *Serratia plymuthica* strain As3-5a(5) achieved 92% Cu(II) biosorption (from an initial concentration of 3.14 mM) within 4 min in a non-proliferating planktonic cell system, and 98% biosorption in a biofilm-based system on sintered glass. Maximum metal biosorption was achieved by late stationary phase grown cells (72 h), likely due to an increased protein fraction in the tightly bound extracellular polymeric substances. When in the presence of real electroplating wastewater containing 40 mM Cu(II) at pH 1.9, planktonic cell system (10^11^ cells mL^−1^) achieved 97% Cu(II) biosorption. These results highlight the strong potential of *Serratia plymuthica* strain As3-5a(5) for developing efficient biological systems for heavy metal removal from industrial wastewater. Furthermore, this work provides valuable insights into sustainable biotechnological approaches for copper remediation, with potential applications in catalytic processes and metal recovery within a circular economy framework. Future studies should involve synthetic biology approach to improve copper sequestration and to investigate the scalability of these systems to higher technology readiness levels under real industrial wastewater conditions.

**Supplementary Information:**

The online version contains supplementary material available at 10.1007/s10532-026-10245-6.

## Introduction

The rapid expansion of industrialization and urbanization has led to the widespread accumulation of heavy metals (HM) in the environment, posing a significant threat to ecosystems and public health (Ahmed et al. 2022). Among these HM, copper is an essential trace element required for various biological processes but becomes toxic at elevated concentrations (Ghoniem et al. 2020). Cu(II) ions are commonly found in wastewaters generated by electroplating, smelting, alloy manufacturing, and battery production industries (Liu et al. 2024). Prolonged exposure to Cu(II) has been associated with liver and kidney damage, respiratory issues, anemia, and hepatocellular toxicity. Consequently, the World Health Organization has established a guideline value of 0.03 mM for Cu(II) in drinking water (Demarco et al. 2023).

Bacterial extracellular polymeric substances (EPS) play a crucial role in biosorption by providing active binding sites for metal cations through functional anionic groups such as carboxyl, hydroxyl, and amino groups (Zadeh et al. 2023). EPS form a structured protective layer around microbial cells, enhancing their ability to withstand environmental stressors (Liu et al. 2024). It is typically classified into loosely bound EPS and tightly bound EPS based on its association with the cell surface. Composed primarily of proteins, polysaccharides, nucleic acids, lipids, and humic substances, EPS can account for up to 90% (w/w) of the organic matter in biofilms. Studies have shown that proteins exhibit a higher affinity for Cu(II) adsorption than polysaccharides, with protein-associated carboxyl groups playing a crucial role in metal binding (Liu et al. 2024). Moreover, extracellular binding of Cu(I), Cu(II) or both can occur via periplasmic CopC/PcoC family cuprochaperones (Udagedara et al. 2019), via the membrane-associated CopL (Rosario-Cruz et al. 2019) or as extracellular copper nanoparticles (Pantidos et al. 2018). The importance of studying copper-accumulating strains resides in the possibility to recover metal from biomass or the use of copper-organic complexes as biocatalysts (Yang et al. 2020; Gandolfi et al. 2022). Among bacterial species documented for EPS production (Kumar et al. 2019) and HM biosorption (Díaz et al. 2022), *Serratia plymuthica* strain As3-5(5a) was previously reported to remove Cu(II) up to 91.5% from a 3.14 mM aqueous solution with a maximum biosorption capacity of 80.5 mg g⁻^1^, and Ni(II) up to 89.4% from a 0.85 mM solution with a biosorption capacity of 33.5 mg g⁻^1^ (Zanetti et al. 2022). *S. plymuthica* is classified as a Biological Risk Group 2 agent that displays high genomic plasticity and ecological adaptability (Silva et al. 2025). Since potential pathogenicity might limit the exploitation of wild strains, synthetic biology could offer an opportunity to develop safe and efficient HM removing recombinant strains to be used in contained environments (Giachino et al. 2021).

Several technologies have been developed to remove HM from the environment, including chemical precipitation, ion exchange, membrane technology, and solvent extraction. However, these conventional methods often suffer from limitations such as low efficiency, high operational costs, and generation of toxic sludge. In contrast, biosorption has emerged as a promising alternative for HM removal due to its cost-effectiveness, simplicity, and environmental sustainability (Liu et al. 2024). Planktonic cells differ from biofilm cells in terms of surface properties and EPS organization. They interact with dissolved organic matter, contributing to key ecological processes (Jenkinson et al. 2021). Compared to planktonic systems, biofilm-based systems have gained attention for wastewater treatment, due to their high microbial biomass density, improved immobilization capacity on microporous microcarriers, and enhanced metal biosorption efficiency (Priyadarshanee et al. 2021).

Although previous studies have examined EPS-mediated Cu(II) biosorption, the specific EPS fractions of *Serratia* involved in metal binding, particularly using electroplating wastewaters, remain poorly characterized. This study addresses this gap by explicitly linking EPS composition to biosorption performance in both planktonic and biofilm systems, and by evaluating the surface properties of *S. plymuthica* strain As3-5a(5), known for its Cu(II) biosorption capacity. Additionally, the ability of *S. plymuthica* As3-5a(5) to form biofilms on microporous microcarriers was evaluated to assess its potential for application in real wastewater treatment. Given the limited knowledge of how distinct EPS fractions contribute to Cu(II) binding and the lack of data on *S. plymuthica* biofilm systems in real electroplating wastewater, this study is essential to bridge the gap between laboratory observations and the development of scalable, sustainable bioremediation strategies.

## Materials and methods

### Chemicals and reagents

Chemicals and reagents were analytical grade and used directly in the experiments. Luria–Bertani (LB) medium was obtained from VWR BDH Chemicals (Pennsylvania, USA). Copper chloride dihydrate (CuCl₂·2H₂O, ≥ 99%, Sigma-Aldrich, St. Louis, MO, USA) was prepared as a 3.14 mM solution (pH 5.03) in Milli-Q water. 1,10-Phenanthroline (≥ 99%, Sigma-Aldrich) was used as received. Phosphate–potassium (PK) buffer (1 M, pH 7.2) and phosphate-buffered saline (PBS, 1X, pH 7.2) were freshly prepared. Concanavalin A (ConA)–Alexa Fluor™ 488 conjugate was purchased from Invitrogen (Waltham, MA, USA). SYTO 9 and propidium iodide (PI) from the BacLight™ viability kit were obtained from Invitrogen (Waltham, MA, USA). Deuterium oxide (D_2_O, 99.9 atom % D, Sigma-Aldrich) was used for spectroscopic analysis. Glutaraldehyde solution (25%, Electron Microscopy Sciences, Hatfield, PA, USA) was diluted in 0.1 M cacodylate buffer (pH 7.2) prior to use. Osmium tetroxide (OsO_4_, 2% aqueous solution, Electron Microscopy Sciences) was employed for sample fixation. Ethanol (EtOH, ≥ 99.8%, Sigma-Aldrich) was used in dehydration steps. Crystal violet (0.1% w/v, Sigma-Aldrich) was used for staining assays.

### Bacterial growth conditions

The *S. plymuthica* strain As3-5a(5) (NCBI PP291912) used in the present study is able to resist to the presence of Cu(II) 8 mM, as previously determined in minimal mineral medium (Zanetti et al. 2022). The strain was cultivated in LB medium and is preserved as glycerol stocks in the AgEM-Lab collection at the Department of Food, Environmental and Nutritional Sciences (DeFENS) at the University of Milan.

### Extracellular polymeric substances extraction, quantification and characterization

The influence of incubation time and cell washing on EPS layers characterization was assessed. *S. plymuthica* strain As3-5a(5) cells were harvested at the exponential (24 h) and stationary (48–72 h) growth phases by centrifugation (10,000 × *g* for 20 min at 4 °C). Previous results (Zanetti et al. 2022) showed that strain As3-5a(5) reached the stationary phase after 48 h incubation and that Cu(II) biosorption was higher at the late stationary phase (72 h). The cells were then subjected to one of the following treatments: a) unwashed, b) water-washed (MilliQ water), or c) buffer-washed (PK buffer followed by MilliQ water). Cells were resuspended (7.43 × 10^10^ cell mL^−1^) at room temperature by orbital agitation at 100 × *g*. To minimize potential interference from the growth medium, the cell biomass was washed with water and phosphate buffer to expose functional groups on the cell surface. Buffer washing facilitates the detachment of soluble proteins and loosely bound molecules from the cell envelope, thereby making previously blocked or masked functional groups accessible. Microscopy and Live and Dead (BacLight^TM^ viability kit) cell count assured that cell integrity was maintained.

EPS extraction and quantification from strain As3-5a(5) was performed in triplicate. EPS was fractionated into loosely bound EPS and tightly bound EPS following the protocol described by Zanetti et al. (2022). Briefly, 3 g of wet biomass (unwashed, water-washed and buffer-washed) was resuspended in phosphate-buffered saline (PBS). The suspensions were subjected to ultrasonication at 40 kHz and 30 W for 30 s (Ultrasonic Processor, Hielscher Ultrasound Technology, Teltow, Germany) and put on ice after treatment, followed by centrifugation (4000 g for 15 min) to isolate the loosely bound EPS fraction while maintaining cell structure. The resulting pellet was resuspended in PBS, thermally treated at 70 °C for 30 min, and centrifuged to obtain the tightly bound EPS fraction without cell lysis (Baptista et al. 2022). Polysaccharide content of each fraction was quantified using the anthrone blue-green method with a glucose standard (Dreywood 1946), while protein concentration was measured using the Bradford assay (Bradford 1976).

Finally, Spearman and Pearson correlation analyses were conducted to evaluate the interaction between specific Cu(II) biosorption (mg g⁻^1^) and EPS protein and polysaccharide content (mg g⁻^1^ dry weight) at 24, 48, and 72 h of incubation, across unwashed, water-washed, and buffer-washed cells (Zecchin et al. 2023).

### *In silico* analysis of extracellular polymeric substances

An *in silico* approach was applied to elucidate the molecular interactions between specific monosaccharide components of the EPS from *S. plymuthica* and the lectin protein ConA, followed by experimental quantification of cell viability using flow cytometry. ConA is a homotetrameric lectin, with each subunit containing a single carbohydrate-binding site capable of recognizing α-D-mannose and α-D-glucose, with a preferential affinity for mannose (Dani et al. 1981). The pipeline included retrieving the 3D structure of ConA from the Protein Data Bank (PDB) and monosaccharide structures from public chemical databases (ChEMBL, PubChem, ChemSpider). The protein structure was processed using ChimeraX to remove non-standard residues (Pettersen et al. 2021). Binding pockets were predicted using Kdeep (Jimenez et al. 2018), COACH-D (Wu et al. 2018), and ProteinPlus (Fährrolfes et al. 2017). Molecular docking was performed using CB-Dock2 (Liu et al. 2022) and SwissDock (Roy et al. 2020) to estimate binding affinities (ΔG), assess the stability of the complexes and identified the optimal docking poses, representing the most energetically favorable 3D orientation of the monosaccharide when bound to the protein. PDBsum was subsequently used to analyze the protein–ligand interactions, identifying hydrogen bonds, non-bonded contacts, and the specific amino acid residues involved (Laskowski et al. 2018; Fig. S1). A higher number of non-bonded contacts relative to hydrogen bonds typically indicates that hydrophobic and van der Waals interactions play a dominant role in stabilization. In later experimental step, flow cytometry confirmed that most exhibited detectable levels of mannopyranosyl and α-glucopyranosyl residues.

### Detection of extracellular polymeric substances by flow cytometry analysis

Flow cytometry was conducted to quantify live and dead cells and to evaluate the presence of a polysaccharide EPS layer, which may contribute to Cu(II) biosorption. *S. plymuthica* strain As3-5a(5) cells were grown in LB media and harvested after 24, 48, and 72 h of incubation. Prior to analysis, the cell suspension was adjusted to 7.43 × 10^10^ cells mL^−1^, and cells were either left unwashed or buffer-washed. Sample preparation for flow cytometry analysis was carried out as described by Bancalari et al. (2022). Staining was performed using ConA-Alexa Fluor ® 488 Conjugate which selectively binds to mannopyranosyl and a-glucopyranosyl residues to detect EPS present on the cell surface. ConA (7.5 mg mL^−1^) was added to 1 mL of culture, opportunely diluted in 0.45 mm filtered distilled water, mixed and incubated for 15 min at room temperature in the dark prior to analysis. SYTO9 and propidium iodide (PI) from BacLight^TM^ viability kit were used to stain live and dead cells, respectively, following the manufacturer’s instructions to quantify cell density. Each staining procedure was conducted in triplicate. All samples were analyzed using the flow cytometer with the following threshold settings: FSC 3,000; SSC 1000; and 50 mL collected. SYTO9 (excitation 488 nm, emission 530/30 nm) and Alexa Fluor^®^ (excitation 488 nm, emission 519 nm) fluorescence intensities were recovered in the FL1 channel. PI fluorescence (excitation 488 nm, emission filter 630/30 nm) intensity was recovered in the FL3 channel. Data were collected as logarithmic signals, with an event rate typically less than 2000 events/s. The acquired data were analyzed using BD Accuri™ C6 software (version 1.0.; BD Biosciences, Milan, Italy).

### Cu(II) biosorption experiments: Planktonic cell system

*S. plymuthica* cells were treated as described in the previous paragraph. Optimal biomass and Cu(II) concentrations and cell-metal contact time were in accordance with the best conditions retrieved previously (Zanetti et al. 2022). Cells were pelleted by centrifugation (10,000 × *g* at 20 °C for 10 min). The resulting biomass was resuspended in 4 mL of a 3.14 mM CuCl_2_ solution (pH 5.03) prepared in MilliQ water and cell suspension was standardized at 7.43 × 10^10^ cells mL⁻^1^ (10.87 log cell mL⁻^1^). After an initial contact time of 4 min, 600 µL aliquots of the suspension were centrifuged for 3 min at 10,000 × *g* to separate the adsorbent biomass from the supernatant. Cu(II) concentration in the supernatant was measured using the 1,10-phenanthroline–copper UV–Vis spectrophotometric method (Pallenberg et al. 1995) and confirmed by inductively coupled plasma–mass spectrometry (ICP-MS). All experiments were performed in triplicate, and abiotic controls were analyzed in parallel to account for non-biological Cu(II) adsorption.

### Determination of extracellular polymeric substances proton relaxation time

The Low-Field Nuclear Magnetic Resonance (LF-NMR) proton relaxation time measurements were performed by a Spinsolve 1.5 LF-NMR spectrometer (Magritek Ltd.) with operating resonance frequency at 60.0 MHz.

T1 measurements were conducted using an inversion recovery pulse sequence. 2 scans of 1.6 s acquisition time, repetition time of 15 s, with 21 steps were taken for the T1 measurements. The structure of a biofilm can influence relaxation rates by limiting the rotational mobility of protons. This restriction occurs in various biofilm components such as the EPS matrix and cell clusters. The chemical environment of a biofilm also affects relaxation, through mechanisms such as chemical exchange between protons in water and functional groups on large EPS molecules. Polysaccharides, particularly gel-forming polysaccharides, often provide structure to biofilms [and may strongly affect T1 and T2 relaxation rates by restricting rotational motion (Willett et al. 2024).

4 scans with an acquisition time of 0.4 s, 2 s repetition time, 20 steps with a Carr-Purcell-Meiboom-Gill (CPMG) echo time of 0.5 ms were taken for the T2 measurements. The distributed multi-exponential fitting of CPMG decay envelope curves and the calculation of T2 values were performed by using Inverse Laplace analysis.

High-Resolution Magic Angle Spinning Nuclear Magnetic Resonance (HRMAS NMR) diffusion measurements ^1^H HR-MAS NMR spectra were acquired on an FT-NMR Avance^TM^ 500 (Bruker BioSpin GmbH, Rheinstetten, Germany) with a superconducting ultrashield magnet of 11.7 T (^1^H frequency: 500.13 MHz) located in University of Milano (COSPECT). HRMAS NMR spectra were acquired using a sample spinning rate of 5 kHz. Spectra were acquired at 30 ◦C (± 0.1 ◦C). The temperature was under control of the BVT 3000 Unit (Bruker BioSpin GmbH, Rheinstetten, Germany). For HRMAS NMR approximately 50 μL of sample was inserted into a Kel-F disposable insert for a 50 μL volume and subsequently in a 4 mm MAS ZrO_2_ rotor (Bruker). 10 μL of deuterium oxide (D_2_O) was added. DOSY were acquired using the pulse-program ‘ledbpgppr2s’, Δ ranging from 100 to 600 ms; gradient pulse: 2.5 ms; number of increments: 64. For each experiment, 24 scans were acquired. The pulse gradients were incremented from 2 to 95% of the maximum gradient strength in a linear ramp. Raw data were processed using the standard DOSY software present in the Bruker library (TOPSPIN v. 1.3). Samples of commercially available dextrans with different molecular weights were used to build the calibration curve (D = −38317ln(Mw)−491,270 (R^2^ = 0.998), Dextran 9-11 KDa, Dextran 35-45 KDa and Dextran 145–150 KDa. The molecular weight was calculated from the exponential equation of the calibration curve.

### Morphological observation and copper localization

Scanning electron microscopy (SEM) was performed to investigate the morphology of *S. plymuthica* following the binding of EPS to Cu(II). Before analysis, the buffer-washed biomass was adjusted to a concentration of 7.43 × 10^10^ cells mL^−1^. Cells were exposed to a Cu(II) solution (3.14 mM) for 4 min, harvested at 24 and 72 h of growth, and resuspended in 1 mL of 2.5% glutaraldehyde (dissolved in 0.1 M cacodylate buffer, pH 7.2). The suspensions were centrifuged (10,000 × *g* for 10 min) and washed three times in cacodylate buffer (0.1 M, pH 7.2). The samples were then resuspended in 2% osmium tetroxide (OsO_4_, dissolved in H_2_O) for 1.5 h at 4 °C in the dark and progressively dehydrated in ethanol (EtOH) from 25 to 100%. After dehydration, samples were sputter-coated with gold using a High Vacuum Coater (Leica Microsystems, Wetzlar, Germany). Observations were carried out with Leo 1430 microscope (Zeiss) equipped with energy-dispersive spectroscopy (EDS).

### Cu(II) biosorption experiments: biofilm-activated carries

Strain ability to form biofilm and to adsorb Cu(II) on microcarriers was evaluated using the crystal violet assay in 96-wells plates. According to Allkja et al. (2021) and Peeters et al. (2008), 200 μL of LB medium were added to each well in triplicate. Briefly, half of the plate was inoculated with 0.5% (v/v) of a 24-h pre-inoculum, while the other half served as an abiotic control. Cell turbidity was monitored at 24-, 48- and 72-h incubation, using a microtiter plate reader (OD_600_; BioTek, Synergy LX, multimode reader, Winoosky, VT, USA). The supernatant was discarded, and the wells were washed with PBS. The biofilm was fixed by incubation with 200 μL of 99–100% for 15-min at room temperature. After removing the ethanol, the plates were air-dried. Subsequently, 200 μL of 0.1% crystal violet solution was added to each well, and the plates were incubated for 15 min at room temperature. The crystal violet was removed, and the wells were washed three times with sterile distilled water. Finally, 200 µL of 99–100% ethanol was added to each well, and the plates were incubated for 30 min at room temperature, with shaking at 125x*g*. Absorbance was measured at 595 nm.

Cu(II) biosorption from a 3.14 mM solution by bacterial biofilms was evaluated using various microporous microcarriers as growth substrates: sodium alginate beads (Duarte et al. 2013), gardening substrate beads (Agrilit 3, Agriperlite, Perlite Italiana s.r.l.) and sintered glass beads (Amtra^®^, glax stone ultra). The carriers were incubated in 20 mL LB medium inoculated with 0.5% (v/v) of a 24-h pre-inoculum. After 72 h of incubation, the carriers were harvested, buffer-washed, and exposed to a 3.14 mM Cu(II) solution. Cu(II) biosorption was measured after 4 min contact using the 1,10-Phenanthroline–copper UV-Vis spectrophotometric method (Pallenberg et al. 1995). The biosorption capacity of the bacterial cells for Cu(II) was calculated by measuring the difference in metal concentration before and after adsorption. All adsorption experiments on activated microcarriers were performed in triplicate, with abiotic controls included.

### Cu(II) biosorption experiments from electroplating wastewaters

Biosorption experiments were performed using three real electroplating wastewater samples collected from the rinsing tanks of an electroplating facility. Prior to use, the samples were characterized for elemental composition and pH using ICP-MS (Table S1). Wastewater 1 corresponded to the first rinsing stage, occurring in the absence of an electrolytic treatment, and therefore containing the highest Cu(II) concentration. Wastewater 2 was collected after the electrolytic treatment step, where part of the Cu(II) is removed through electrochemical deposition, resulting in a lower residual concentration. Finally, Wastewater 3 was sampled from a secondary rinsing stage, where the combined effects of additional rinsing and prior Cu(II) removal steps resulted in the lowest metal concentration.

The wastewaters were treated using both planktonic cells and biofilm formed on sintered glass. Cell viability was determined by plate count method. Cu(II) biosorption was measured using ICP-MS and 1,10–phenanthroline–copper UV-Vis spectrophotometric method (Pallenberg et al. 1995). Each experiment was conducted in triplicate, with abiotic controls included in all analysis.

### Statistical analysis

All experiments were performed in triplicate. Significant differences between groups were assessed using one-way ANOVA followed by Tukey’s post hoc test (p ≤ 0.05) for: Cu(II) biosorption by cells incubated 24, 48, and 72 h under different washing treatments; Cu(II) biosorption after contact with microporous microcarriers activated by *S. plymuthica* strain As3-5a(5); and Cu(II) biosorption from electroplating wastewaters in planktonic and biofilm systems. Spearman and Pearson correlation analyses were performed to evaluate relationships between Cu(II) biosorption and EPS protein and polysaccharide content (Zecchin et al. 2023). All analyses were conducted using R software (version 4.4.3, R Foundation for Statistical Computing, Vienna, Austria).

## Results and discussion

### Extracellular polymeric substances characterization and quantification

The molecular weight of the EPS of *S. plymuthica* strain As3-5a(5) ranged from 16 to 43 kDa as estimated by diffusion-ordered spectroscopy (DOSY), indicating a heterogeneous mixture of biopolymers.

To better understand EPS development, cells were harvested at different growth stages and subjected to washing treatments of increasing ionic strength (*i.e*., unwashed, water- and buffer-washed). Analysis of EPS at each incubation time revealed that 41% of the total EPS (Table S2) was loosely bound, while 59% remained tightly associated with the cells. These results suggest the existence of EPS layers with varying binding strengths, some of which can be removed more easily. Increasing ionic strength preferentially released loosely bound layers, thereby enriching the proportion of tightly bound EPS and confirming the presence of layers with different binding strengths surrounding the cells (Table 1). This stratified organization of EPS is consistent with the presence of an outer, more soluble or weakly attached layer that can act as a readily exchangeable interface with the environment, and an inner, more compact and resilient layer that remains tightly associated with the cell surface. Such a multilayered arrangement has been proposed to provide functional advantages, with the outer layer mediating initial interactions, nutrient or ion exchange, and protection against sudden environmental stress, while the inner layer ensures structural integrity and sustained attachment to the cell envelope (Liu et al. 2024).

**Table 1:** EPS characterization of total, loosely bound (LB) and tightly bound (TB) polysaccharides and proteins (mg g^−1^ d.w.) of *Serratia plymuthica* strain As3-5a(5) (Mean ± SD, n = 3)

Cell treatment	Incubation time (h)	EPS-Polysaccharides (mg g^−1^ d.w.)			EPS-Proteins (mg g^−1^ d.w.)		
		Total	LB	TB	Total	LB	TB
Unwashed	24	25.58 ± 11.92	1.95 ± 0.10	23.63 ± 1.70	23.51 ± 4.29	7.84 ± 0.47	15.67 ± 0.01
	48	21.38 ± 5.41	5.79 ± 0.49	15.59 ± 0.89	24.49 ± 5.45	17.19 ± 0.78	7.30 ± 0.65
	72	36.62 ± 1.9	16.72 ± 0.65	19.90 ± 1.04	48.67 ± 11.78	35.10 ± 0.58	13.58 ± 0.08
Water-washed	24	44.76 ± 0.5	3.79 ± 0.15	40.97 ± 0.35	30.09 ± 0.87	13.89 ± 0.63	17.01 ± 0.24
	48	76.84 ± 9.07	20.84 ± 3.89	56.00 ± 5.69	64.63 ± 1.99	47.72 ± 1.04	16.91 ± 0.95
	72	44.78 ± 2.02	14.77 ± 0.94	30.01 ± 1.09	42.6 ± 0.37	28.30 ± 0.12	14.30 ± 0.25
Buffer-washed	24	27.54 ± 0.56	4.46 ± 0.37	23.08 ± 0.19	22.1 ± 0.66	15.27 ± 0.52	6.83 ± 0.14
	48	32.29 ± 0.96	2.19 ± 0.09	30.10 ± 0.87	21.45 ± 1.68	11.28 ± 1.07	10.17 ± 0.61
	72	24.81 ± 2.46	1.81 ± 0.18	23.00 ± 2.28	30.37 ± 1.18	15.88 ± 0.57	14.49 ± 0.61

Polysaccharides accounted for 52% of the total EPS, 22% of which were in the loosely bound fraction and 78% in the tightly bound fraction, indicating a higher abundance in the tightly bound layers. In 72 h cells, total EPS and loosely bound polysaccharides decreased after buffer washing, whereas tightly bound polysaccharides were more evenly distributed. Proteins were distributed between loosely (62%) and tightly bound (38%) fractions. At 72 h, tightly bound proteins represented 57%, 48%, and 55% of the total EPS across the different washing treatments, respectively. Notably, protein levels peaked at 72 h in the buffer-washed samples, reaching 14.49 mg g⁻^1^ d.w..

Flow cytometry results (Table 2) provided additional insights into the dynamics of sugar-containing EPS over time. In unwashed cells, mean fluorescence intensity (MFI, FL1-H) decreased from 24 to 72 h, coinciding with a reduction in cell viability after 24 h (Table S3). Interestingly, the percentage of EPS positive cells remained high at all time points (≥ 89–100%) indicating that most cells retained detectable mannopyranosyl and α-glucopyranosyl residues. After buffer washing, fluorescence intensity decreased markedly at all time points, suggesting the removal of soluble and loosely bound EPS polysaccharides. Washing may also have affected cell integrity, resulting in increased cellular autofluorescence. As reported by Renggli et al. (2013), such autofluorescence can partially overlap with or mask the ConA signal, reducing the apparent EPS-associated fluorescence detected by flow cytometry. Nevertheless, EPS characterization (Table 1) confirmed a decrease in polysaccharides after buffer-washing at 72 h.

**Table 2:** Effect of buffer washing on the detection of extracellular polymeric substances (EPS) positive cells by flow cytometry at different incubation times, expressed as percentage (%) on total fluorescent units and mean of fluorescence intensity (MFI) (Mean ± SD, n = 3)

	Incubation time (h)	EPS positive cells (%)	MFI (FL1-H)
Unwashed	24	89	6779 ± 203
	48	100	4335 ± 635
	72	92	3764 ± 53
Buffer-washed	24	100	343 ± 23
	48	100	214 ± 49
	72	96	369 ± 90

### Copper biosorption: Planktonic cell system

**Fig. 1:**
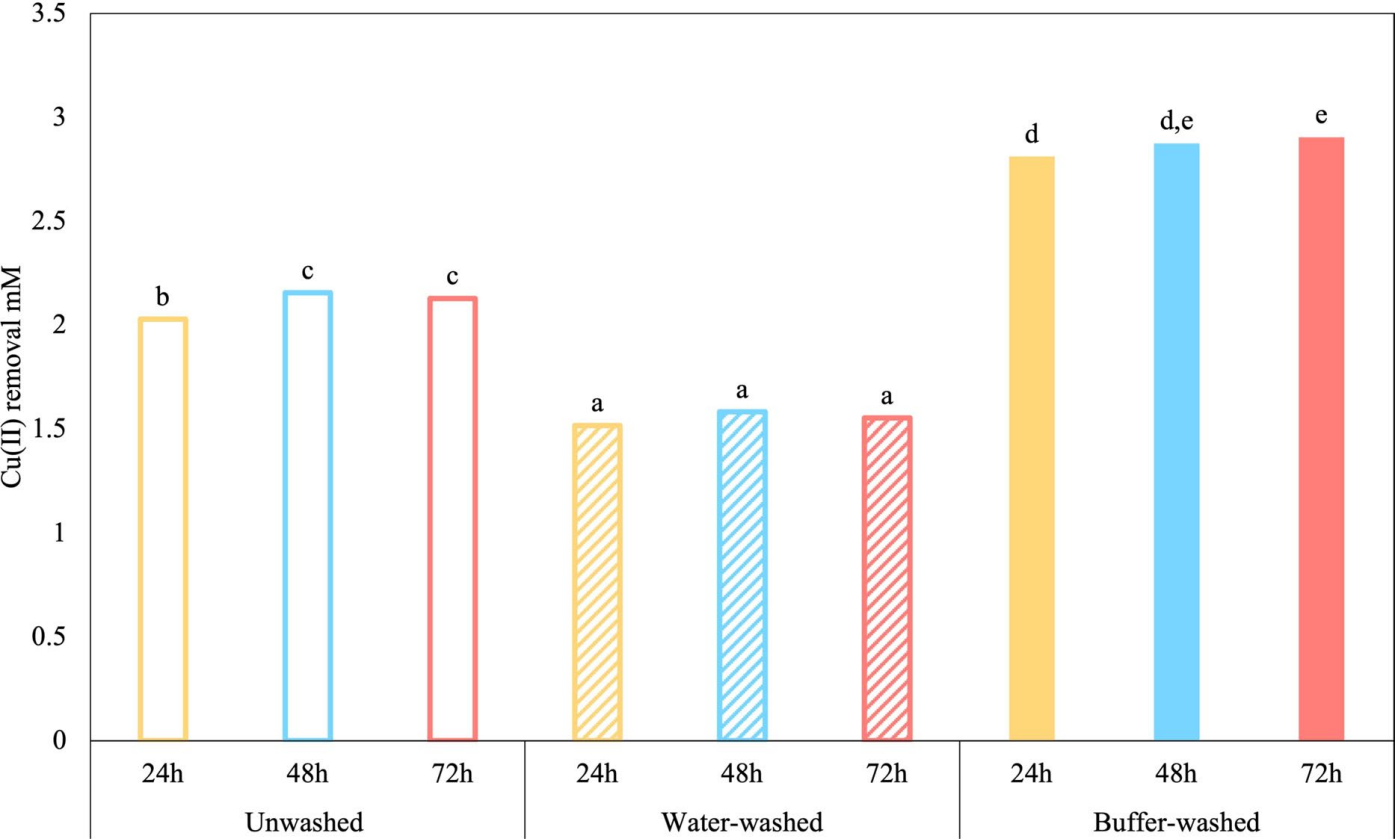
Cu(II) biosorption (initial concentration 3.14 mM; pH 5.03) measured on cells incubated for 24-, 48-, and 72-h and subjected to different washing treatments (unwashed, water- and buffer-washed). Statistical comparisons were performed across all treatments and time points using one-way ANOVA followed by Tukey’s test (p ≤ 0.05)

Biosorption experiments using 3.14 mM Cu(II) were performed with *S. plymuthica* strain As3-5a(5) cells under different washing treatments. Buffer-washed cells achieved the highest Cu(II) biosorption at 92% (2.89 mM), followed by unwashed cells (68%) and water-washed cells (49%) (Fig. 1). The higher biosorption observed in unwashed with respect to water-washed cells suggests that negatively charged sugars or peptides of LB medium might have enhanced biosorption process, consistent with Ahamed et al. (2024). Copper biosorption was higher in late exponential and stationary phase cells (91% and 92%, respectively), indicating that changes in EPS composition during growth influence metal-binding efficiency. This was particularly evident in buffer-washed cells which contained EPS with a higher protein-to-polysaccharide ratio (Table 1). This finding aligns with Ciani et al. (2024), who reported that excessive EPS amounts can reduce metal-binding capacity by limiting access to active sites. A subsequent washing step with PK buffer may have released cations tightly bound to EPS functional groups, enhancing metal binding and improving biosorption efficiency. This is consistent with Tahir et al. (2019), who demonstrated that, while EPS are essential for metal biosorption, excessive accumulation on cell surfaces can hinder the process by obstructing active binding sites.

Furthermore, the pH of the solution emerged as a strong factor influencing Cu(II) biosorption efficiency. In fact, in a CuCl₂ solution at pH 4.42, biosorption reached 43%, whereas at pH 1.9, only 21% of Cu(II) was removed. This emphasizes the need to consider pH as a critical parameter when designing and optimizing biosorption processes.

In addition, desorption experiments with HNO_3_ 0.1 M led to the release of approximately 60% of the adsorbed Cu(II), indicating that a substantial fraction of the metal is bound externally but through interactions of different strength. Nevertheless, the incomplete release also suggests that part of the Cu(II) may remain more stably associated with the cell surface or even be internalized (Lo et al. 2003). Further analysis will address this issue.

### Differentiation of water states of extracellular polymeric substances

To investigate how EPS structure and Cu(II) ions influence water distribution and mobility, LF-NMR relaxation measurements (T1 and T2) were performed. This analysis aims to distinguish and quantify the different water populations (strongly bound, vicinal, and interstitial) within the microbial biomass, providing insight into changes in EPS organization and biopolymer cross-linking induced by metal adsorption.

After the application of an excitation pulse, the hydrogen nuclei will relax back to equilibrium on two different time scales: T1 and T2. The T1 (or longitudinal) relaxation time and the T2 (or transverse) relaxation time provide information on the physico-chemical environments in which different water populations exist (Callaghan 1991). T1 relaxation is related to the magnetization return to thermal equilibrium after an excitation pulse. T2 relaxation is related to molecular interactions that take place in the local magnetic field during the measurement. For instance, the hydrogens present in water within EPS can exchange with the hydrogens on the biopolymer and, consequentially, a shortened of T2 is observed. The values can decrease from approximately 2 s in pure water to hundreds of milliseconds or microseconds.

Due to the different interactions of water with the surrounding environment, T2 relaxation time could be a parameter to detect the mobility of protons. The diversity of water states in microbial biomass is based on the different binding strengths among water molecules and solid phase. Chemical exchange of protons between water and hydroxyl groups on polymer chains (Hillis 1996) as well as the presence of rotationally restricted water in EPS enhances T2 relaxation time relative to bulk water. Generally, a long T2 time indicates a weak binding and, consequently, a higher mobility of water; vice versa, a short T2 suggests a tight linkage between water and structure of surrounding materials (Rudi et al.^2008^). The T2 distribution data are summarized in Table 3 for the cells with and without the presence of Cu(II).

**Table 3:** Distribution data of T1 and T2

Samples	T2 A (ms)	Area peak A (%)	T2b (ms)	Area peak B (%)	T1 (ms)
As3-5a(5)	1.68	3.1	84	96.9	274
As3-5a(5) + Cu(II)	–	–	65	100	467

The quantification of water states was based on the cumulative integration of peak area in T2 distribution spectra. The proportion of each peak area in the total peak area represents the relative amount of corresponding water state (Aursand et al. 2008; Gummerson et al. 1979).

The data suggest the existence of two distinct states of protons in As3-5a(5) cells. T2A could be assigned to the fixed water strongly bound to the cells through hydrogen bond (vicinal water) and T2B could be referred to the water physically trapped in the microbial biomass texture by steric hindrance or adsorption (interstitial water), where its state is governate by the micro- and macro-capillary structure. The presence of Cu(II) slightly affects the T2 relaxation time changing the cross-linked biopolymers in the microbial biomass structure and affecting the interaction with water.

The spin–lattice relaxation process, T1, was slightly different in the considered samples but a longer T1 also means greater mobility (T1 of pure water is 3–4 ms). Also, T1 relation times observed in the considered samples showed that water is trapped in the microbial biomass structure.

### Correlation analysis between Copper biosorption and extracellular polymeric substances layers

**Fig. 2:**
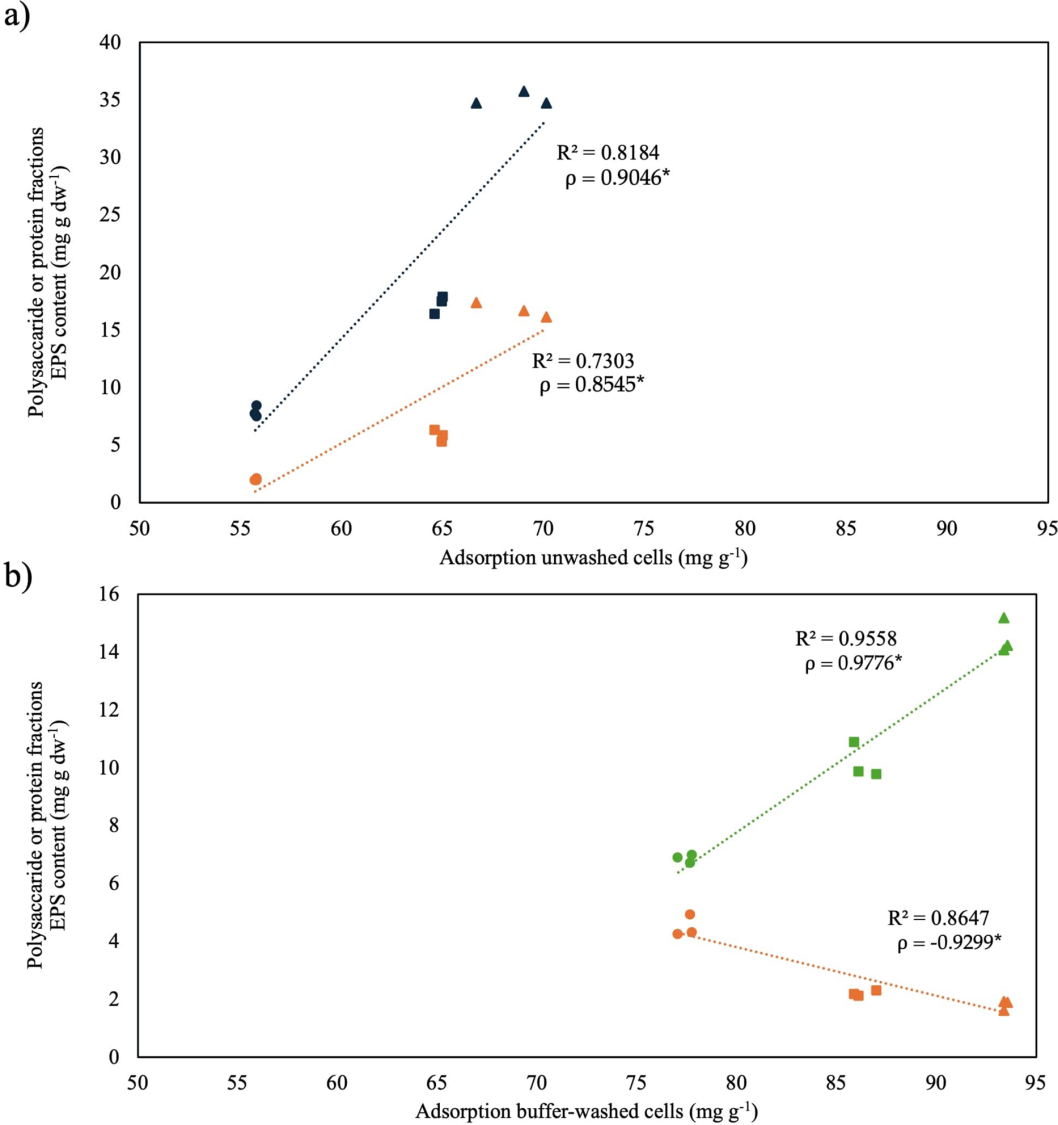
Pearson correlation analysis for **a** unwashed (**a**) and **b** buffer-washed (**b**) cells of loosely bound proteins (blue), tightly bound proteins (green), and loosely bound polysaccharides (orange) and Cu(II) biosorption at 24 (circle), 48 (square) and 72 (triangle) incubation time

Pearson correlation analysis (Table S4, Fig. 2) revealed distinct relationships between Cu(II) specific biosorption and EPS components under different cell-washing conditions. In unwashed cells (Fig. 2a), Cu(II) biosorption correlated positively with both loosely bound proteins (ρ = 0.9046) and polysaccharides (ρ = 0.8545), suggesting their contribution to metal binding in this outer EPS layer. Despite this strong correlation, the overall Cu(II) biosorption was lower compared to buffer-washed cells (Fig. 1). This may reflect the shielding effect of loosely bound polysaccharides, which might limit access to high-affinity binding sites. In buffer-washed cells (Fig. 2b), which exhibited the highest levels of Cu(II) biosorption, a strong positive correlation was observed with tightly bound proteins (ρ = 0.9776), while loosely bound polysaccharides showed a significant negative correlation (ρ =  − 0.9299). This suggests that stronger washing removed interfering outer EPS layers, exposing protein binding sites responsible for efficient metal uptake. No significant correlations were observed in water-washed cells, where biosorption levels were the lowest.

These findings support the idea that proteins, particularly those in tightly bound EPS layer, play a central role in Cu(II) biosorption, whereas loosely bound polysaccharides, although contributing, may act as a physical barrier hindering access to functional groups involved in metal coordination. Previously conducted Fourier Transformed Infrared (FTIR) spectroscopy analysis demonstrated that carboxyl groups of tightly bound EPS proteins are actively involved in Cu(II) binding in *S. plymuthica* strain As3-5a(5) (Zanetti et al. 2022). This hypothesis is supported by Liu et al. (2024) who demonstrated that proteins exhibited significantly higher biosorption capacities for HM, such as Cu(II), Zn(II), and Cd(II), compared to polysaccharides. Two-dimensional correlation spectroscopy further revealed that carboxyl groups in proteins preferentially and more rapidly bind Cu(II) ions than those in polysaccharides (Liu et al. 2024). In accordance with these findings, biosorption in our study was mostly attributable to the protein fraction and occurred within 4 min of contact between cells and metal ions.

### Copper localization on *Serratia plymuthica* strain As3-5a(5)

**Fig. 3:**
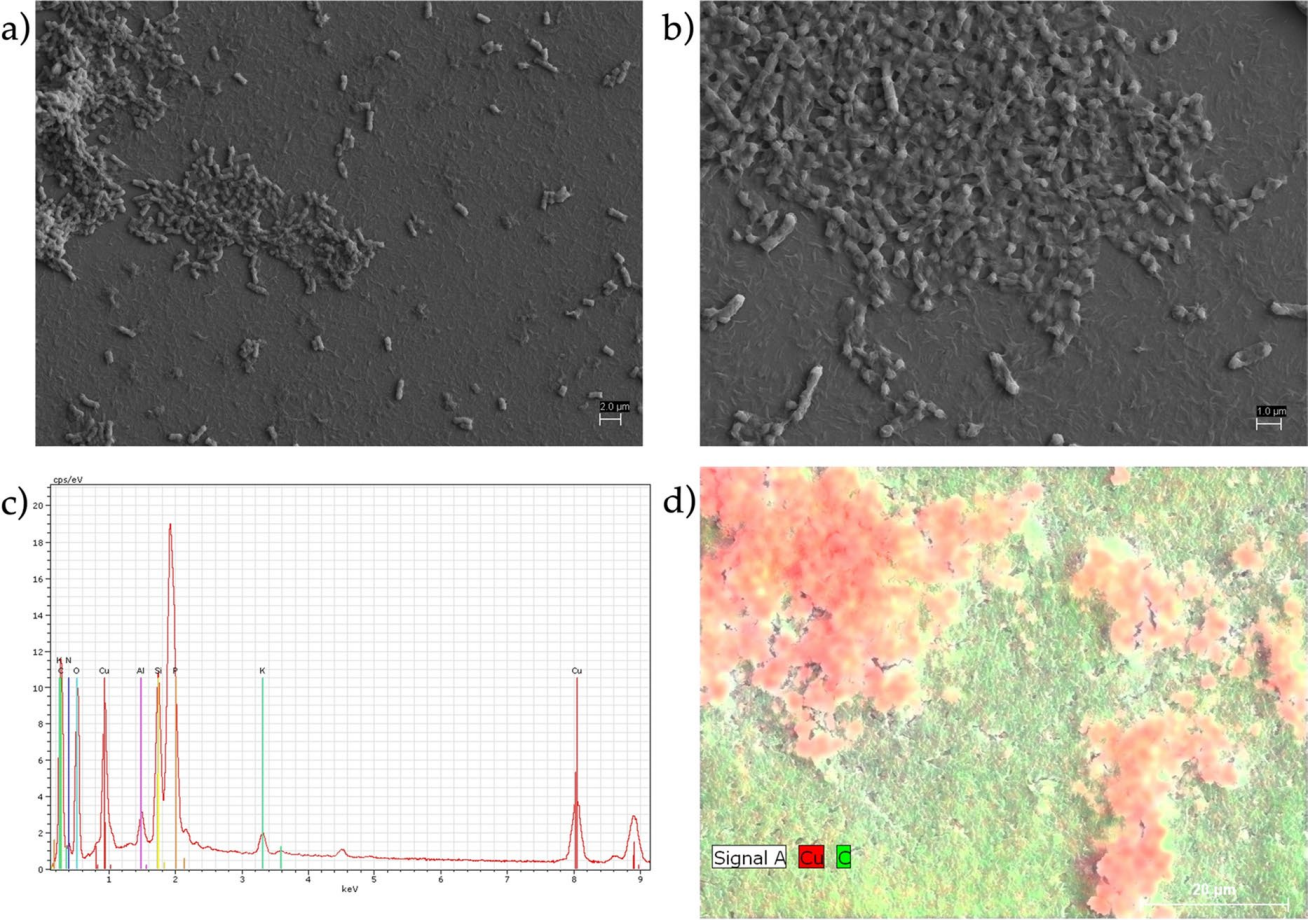
SEM observation of water- and buffer-washed 72 h grown cells of *Serratia plymuthica* strain As3-5a(5) put in contact for 4 min with a 3.14 mM solution of Cu(II) before (**a**) and **b** after (**b**) metal contact. Elemental composition of extracellular polymeric substances detected by EDS (**c**, **d**) peaks of the elements detected and their abundance

Under SEM analysis, *S. plymuthica* strain As3-5a(5) exhibited a rod-shaped morphology, with individual cells measuring 1.5 μm in length and 0.453 μm in width (Fig. S2). SEM images revealed that cells were enveloped by a dense EPS layer, contributing to surface folding and an irregular outer morphology. A slimy material was clearly visible on the cell surface, forming a filamentous polymeric matrix that physically interconnected adjacent rod-shaped cells into a cohesive network (Fig. 3). This observation is consistent with Kumar et al. (2019) who described how initially sparse EPS production evolves into a dense matrix over time, ultimately masking individual cells after 72 h.

To further investigate cell morphology and confirm Cu(II) binding, SEM–EDS analysis was performed on strain As3-5a(5) cells before and after exposure to Cu(II) (Fig. 3a and b). Elemental mapping via EDS (Fig. 3c and d) confirmed the presence of copper on the bacterial surface following biosorption, alongside carbon signals from the organic cellular matrix. During sample preparation, the dehydration step with hexamethyldisilazane (HDMS) caused removal of the EPS layer (Fig. S3). Notably, under these conditions, Cu(II) was no longer detectable on the cell surface, suggesting that the presence of EPS is essential for effective Cu(II) binding. These findings confirm copper presence on *S. plymuthica* strain As3-5a(5) cells and highlight the dual role of EPS in both metal binding and intercellular aggregation.

### Copper biosorption: Biofilm-based systems

To assess the feasibility of using strain As3-5a(5) as microbial biofilter for wastewater treatment applications, three biofilm-supporting carriers were tested: alginate beads, gardening substrate and sintered glass.

Preliminary crystal violet staining demonstrated that strain As3-5a(5) formed more robust biofilms after 72 h of incubation compared to 24 and 48-h time points (Fig. S4), suggesting that older cells are more likely to aggregate and adhere to surfaces due to EPS production. These results are consistent with Peeters et al. (2008) who observed maximum biofilm formation after 72 h incubation and with Guo et al. (2024) who confirmed the ability of *S. plymuthica* strain to form biofilms in microtiter plates, over 5 days.

**Fig. 4:**
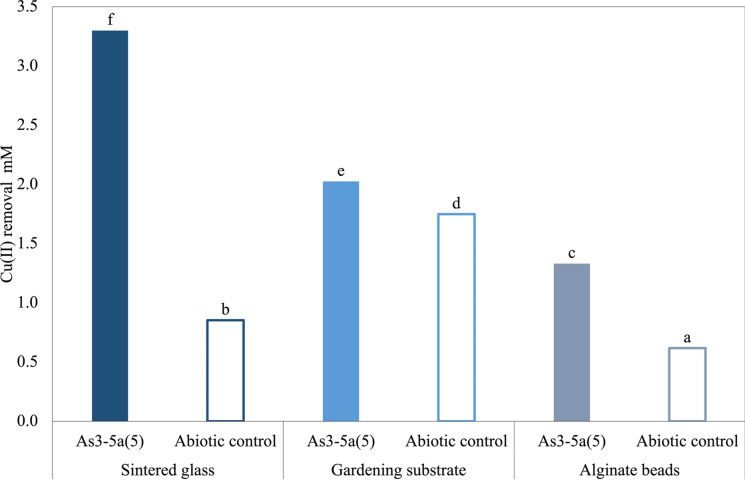
Cu(II) biosorption after four-minute contact with different microporous microcarrier activated by *Serratia plymuthica* strain As3-5a(5) (Tukey’s test, p ≤ 0.05)

The highest Cu(II) biosorption by 72 h-grown biofilms occurred on sintered glass (98%), while the lowest (42%) was observed on alginate beads (Fig. 4). Abiotic controls accounted for 25%, 30%, 19% of Cu(II) biosorption on sintered glass, gardening substrate, and alginate beads, respectively, indicating that part of the biosorption was due to the inherent properties of the carrier materials. Specifically, with the gardening substrate, 8% of Cu(II) removal was attributed to bacterial activity, while 36% was attributed to the material itself.

The ability of strain As3-5a(5) to form biofilm on all tested supports can be attributed to the distinct porosity and surface properties of each material. Sintered glass beads, which achieved the highest Cu(II) removal, possess a wide pore size distribution (10–500 µm) and a high internal surface area (~ 600 m^2^L^-1^), providing extensive surfaces for bacterial adhesion and microenvironments conducive to stable biofilm maturation (Jenjitwanich et al. 2024). The gardening substrate, composed of highly porous expanded volcanic glass (90% total porosity), also supported biofilm formation through its rough surface texture and capillary networks. Additionally, the high porosity and siliceous composition of perlite likely promoted Cu(II) adsorption via surface complexation or ion exchange mechanisms (Lago et al. 2024).

Sodium alginate beads exhibited the lowest removal efficiency, likely due to their smaller average pore size (5–10 nm) and lower mechanical stability, which may limit the development of dense, multi-layered biofilms. Moreover, alginate contains negatively charged functional groups (e.g., carboxylate moieties) that can bind divalent metal ions, contributing to passive metal sequestration (Duarte et al. 2013).

Overall, biofilm formation was more efficient on materials with macroporous structures and rigid matrices, supporting the conclusion that both pore architecture and surface chemistry are key factors in biofilm-based bioremediation. The capacity of biofilms to enhance metal adsorption was further highlighted by Mendrinou et al. (2021), who demonstrated complete removal of Cu(II) (1.5 mM) by biofilm of *Halomonas denitrificans* grown on sintered glass supports. Similarly, Lago et al. (2024) reported that immobilized cells could enhance metal removal efficiency by over 60%, supporting our findings.

In comparison, planktonic cells achieved a Cu(II) biosorption efficiency of 92%, with no significant contribution from abiotic adsorption. However, unlike biofilm-associated systems, planktonic cultures may be less effective in long-term or continuous treatment scenarios, underscoring the importance of stable biofilm carriers for industrial wastewaters bioremediation.

In this study Cu(II) was supplied as CuCl_2_ and experiments were carried out under acidic conditions (pH 5.03), where the free Cu^2^⁺ ion is the predominant species. Therefore, metal speciation was not expected to significantly affect the observed biosorption. However, in more complex environmental matrices, where hydrolyzed or complexed forms of Cu(II) may occur, EPS–metal interactions could be strongly influenced by speciation, as previously shown for other metals such as uranium (Chowdhury et al., 2023).

Although our study focused on biosorption at the cell/EPS level, recent advances in biofilm engineering and synthetic biology offer promising directions for improving metal biosorption. For instance, engineered biofilms with enhanced EPS synthesis or modified EPS composition can provide a higher density of binding sites for metal ions. Moreover, the development of biofilm-based bioreactors and the application of synthetic biology tools (e.g., CRISPR-based modification of EPS biosynthetic pathways) have been shown to increase the efficiency and selectivity of metal biosorption (Mondal et al. 2025). Such strategies could be particularly valuable for scaling up biosorption processes and for integrating biosorption with downstream metal recovery.

### Copper biosorption from electroplating wastewaters: Planktonic cells vs. biofilm

The tested electroplating wastewaters showed markedly different Cu(II) concentrations, ranging from 0.5 mM up to 40.33 mM, reflecting their origin from different rinsing and treatment stages within the galvanic process (Table S1). Their highly acidic pH (≈ 1.2–1.9), due to the addition of acids and process additives, presents a challenging environment for biosorption.

**Fig. 5:**
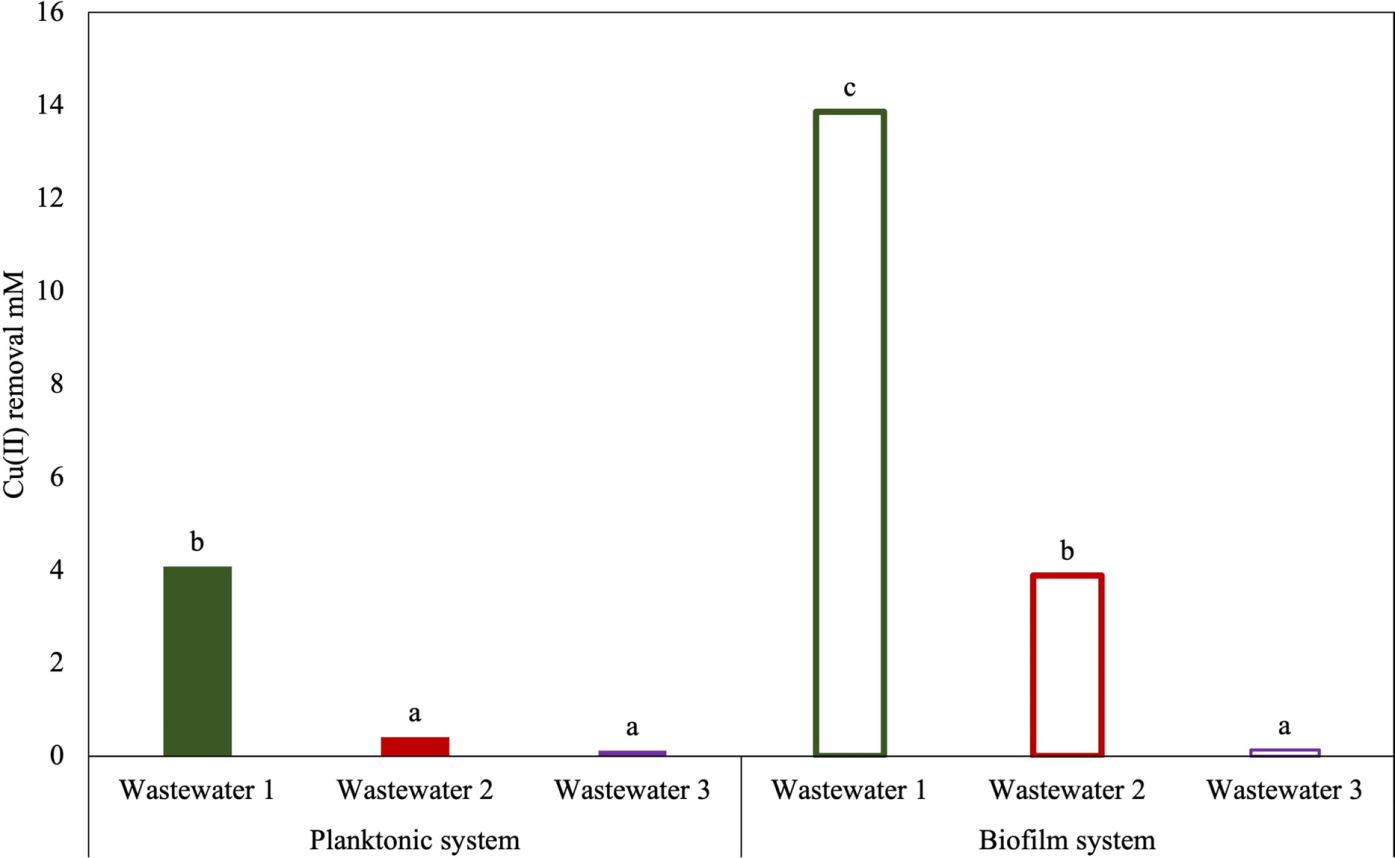
Cu(II) biosorption from electroplating wastewaters in planktonic and biofilm systems (Tukey’s test, p ≤ 0.05)

Cu(II) biosorption capacity of *S. plymuthica* strain As3-5a(5) was evaluated in both planktonic and biofilm-based systems with sintered glass as a support, using 72 h harvested cells. Cu(II) biosorption was significantly higher in the biofilm system, achieving removal efficiencies of 34%, 89%, and 27%, across the three wastewater samples, respectively. In contrast, the planktonic system (7.43 × 10^10^ cells mL^−1^) exhibited lower biosorption efficiencies of 10%, 9%, and 23%, respectively (Fig. 5). Siddharth et al. (2021) similarly reported effective EPS biosorption of multiple HM ions, including Cu(II) (90%), Cr(VI) (88%), Ni(II) (65%), and Pb(II) (73%), from simulated electroplating and synthetic wastewater.

The highest Cu(II) biosorption was observed in the biofilm system with Wastewater 1, which had the highest initial Cu(II) concentration (40.33 mM). Under these conditions, biosorption efficiency improved by 70%, likely due to the combined effects of elevated metal concentration and the favorable structural properties of sintered glass, providing high surface area and a porous matrix for effective bacterial colonization and stable biofilm development. Importantly, the sintered glass biofilm system was demonstrated to be effective even under harsh acidic conditions (pH < 3), consistent with findings by Mendrinou et al. (2021). This robustness is largely attributed to the EPS matrix, which stabilizes the biofilm and facilitates metal binding. Additionally, the porous matrix of supports like sintered glass provides protective microenvironments that shield cells from environmental stressors and inhibitory substances often present in real wastewater, such as surfactants, chelating agents, or competing metal ions.

To enhance Cu(II) biosorption in planktonic cell systems, the biomass concentration was increased. At a bacterial dose of 10^11^ cells mL⁻^1^, Cu(II) biosorption from Wastewater 1 (40.33 mM Cu) reached 97% (residual Cu(II) of 4.08 mM ± 0.24), whereas at 10^10^ cells mL⁻^1^ the biosorption was limited to only 10% (residual Cu(II) of 39.68 ± 0.2). These results highlight the importance of providing a large specific surface area and abundant binding sites to optimize biosorption in environments with high metal concentrations.

Although the results of this study highlight the promising potential of *S. plymuthica* strain As3-5a(5) for Cu(II) removal, it is essential to consider the biosafety implications and ecological risks associated with its large-scale application. *S. plymuthica* is an environmental bacterium that has occasionally been reported as a cause of opportunistic infections in immunocompromised individuals, and it is therefore classified as a Biological Risk Group 2 agent. Moreover, recent evidence indicates that *Serratia* species display high genomic plasticity and ecological adaptability. Silva et al. (2025) demonstrated that plant-associated *Serratia* strains possess diverse traits related to biofilm formation, secondary metabolite production, and horizontal gene transfer, which support their ability to colonize a wide range of environments. While such features may enhance their bioremediation potential, they also raise biosafety concerns, including the risk of environmental dissemination, competition with native microbiota, and opportunistic pathogenicity. Consequently, the application of *S. plymuthica* in wastewater treatment should be preceded by thorough risk assessments and accompanied by mitigation strategies, such as the use of immobilized or inactivated cells. With this respect, the application of a synthetic biology approach might aid in this respect, although the use of genetically modified organisms for bioremediation purposes can pose ethical and legal issues.

## Conclusions

This study demonstrates the central role of the EPS protein fraction in mediating Cu(II) biosorption. Although biofilm systems developed on sintered glass exhibited superior metal removal efficiency, planktonic cells offer practical advantages for large-scale applications due to their simpler cultivation and handling. The high biosorption capacity observed in concentrated planktonic biomass highlights the potential for flexible, hybrid strategies tailored to specific industrial bioremediation needs. However, the presence of complex contaminants and inhibitory substances in real wastewater may reduce the biosorption efficiency of planktonic cells, making biofilm systems a more robust and reliable choice for industrial or environmental remediation contexts. Integrating effective desorption methods could further improve process sustainability by allowing metal reuse. The formation of metal-cell complexes enables effective metal recovery useful for biocatalytic application, aligning with circular economy principles, and for reducing HM levels in at risk populations. Future research should prioritize the optimization of biofilm systems by investigating key parameters such as microcarrier selection, biomass loading, pH control, and metal recovery techniques to maximize performance and industrial applicability.

## Supplementary Information

Below is the link to the electronic supplementary material.

Supplementary file1 (DOCX 1904 KB)

## Data Availability

Data are available in Dataverse (https://dataverse.unimi.it/dataverse/HMBV; 10.13130/RD_UNIMI/SWHMIM).
