# Supplementary material for: Copper biosorption by Serratia plymuthica: crucial role of tightly bound extracellular polymeric substances in planktonic and biofilm systems
Source: Biodegradation. 2026 Jan 16;37(1):25. doi: 10.1007/s10532-026-10245-6 (PMC12811316; doi:10.1007/s10532-026-10245-6)
Supplement: Supplementary file 1 — Supplementary file1 (DOCX 1904 KB) [file 10532_2026_10245_MOESM1_ESM.docx]

**Supplementary materials**

**Copper Removal by *Serratia plymuthica*: Crucial Role of tightly bound EPS in Planktonic and Biofilm Systems**

Alice Melzi^1^, Sarah Zecchin^1^, Milena Colombo^1^, Gigliola Borgonovo^1^, Stefania Mazzini^1^, Subhoshmita Mondal^1^, Stefania Arioli^1^ and Lucia Cavalca^1*^

^1^Dipartimento di Scienze per gli Alimenti, la Nutrizione e l’Ambiente (DeFENS), Università degli Studi di Milano, Via Celoria 2, 20133 Milan, Italy.

*****Correspondence: [lucia.cavalca@unimi.it](mailto:lucia.cavalca@unimi.it)

**Table S1** Electroplating wastewater characterization, metal content expressed in mM determined by ICP-MS (Mean ± SD, n =3).

|  | **pH** | Cu | **Ni** | **Cr** | **Au** | **Zn** | **As** | **Mn** | **Cd** | **Na** | **Mg** | **Al** | **P** | **K** | **Ca** | **Fe** | **Se** |
| --- | --- | --- | --- | --- | --- | --- | --- | --- | --- | --- | --- | --- | --- | --- | --- | --- | --- |
| Wastewater 1 | 1.90 | 40.33±  0.016 | 0.017±  0.2 | 0.019±  0.087 | - | 1.16±  0.06 | 0.04±  0.011 | 0 | 0 | 7.09±  0.06 | 0.12±  0.009 | 0.037±  0.002 | 2.45±  0.14 | 2.48±  0.087 | 262.4±  5.67 | 0.18±  0.01 | 0 |
| Wastewater 2 | 1.20 | 4.39±  0.1 | 0.068±  0.012 | 0.98±  0.046 | - | 8.54±  1.2 | 0 | 0 | 0 | 8.65±  0.13 | 0.29±  0.004 | 0.37±  0.032 | 3.1±  0.98 | 0.56±  0.051 | 73.4±  3.42 | 0.91±  0.043 | 0.013±  0.008 |
| Wastewater 3 | 1.89 | 0.5±  2.227 | 6.39±  0.214 | 4.81±  0.13 | 1.31±  0.096 | - | - | - | - | - | - | - | - | - | - | - | - |

Table S2 Total EPS content (mg g^-1^ d.w. ) of *Serratia plymuthica* strain As3-5a(5) cells collected at different incubation times and treated with increasing ionic strengths (Mean ± SD, n =3)..

| **Cell treatment** | **Incubation period (h)** | **Total EPS**  **(mg g^-1^ d.w.)** |
| --- | --- | --- |
|  |  |  |
| Unwashed | 24 | 49.10 ± 2.24 |
|  | 48 | 45.86 ± 1.24 |
|  | 72 | 85.28 ± 1.62 |
| Water-washed | 24 | 75.66 ± 0.34 |
|  | 48 | 141.47 ± 6.03 |
|  | 72 | 87.38 ± 1.19 |
| Buffer-washed | 24 | 49.65 ± 0.50 |
|  | 48 | 53.75 ± 2.51 |
|  | 72 | 55.17 ± 1.41 |

**Table S3** Distribution (%) of TFU, AFU, and non-AFU populations in *S. plymuthica* As3-5a(5) after live/dead staining, measured by flow cytometry. Data represent mean ± SD (n = 3).

|  | Incubation period (h) | Total Fluorescent units (TFU)  Events mL^-1^ | Mean Arbitrary Fluorescence Unit (AFU) % | Mean not Arbitrary Fluorescence Unit (notAFU) % |
| --- | --- | --- | --- | --- |
| Unwashed | 24 | 1.9E+10±5.8E+09 | 97 | 3 |
|  | 48 | 1.4E+10±1.3E+09 | 62 | 26 |
|  | 72 | 1.6E+10±1.1E+09 | 73 | 38 |
| Buffer-washed | 24 | 7.2E+09±2.5E+08 | 74 | 18 |
|  | 48 | 8.1E+09±1.4E+09 | 82 | 27 |
|  | 72 | 8.8E+09±9.7E+08 | 71 | 23 |

**Table S4** Spearman correlation values of each Cu(II) specific adsorption, EPS content and polysaccharides and protein content ratio.

| **Cu(II) specific adsorption** | **Polysaccharides (mg g^-1^ d.w.)** | | **Proteins (mg g^-1^ d.w.)** | | **Polysaccharides/Proteins** |
| --- | --- | --- | --- | --- | --- |
|  | **LB-EPS** | **TB-EPS** | **LB-EPS** | **TB-EPS** |  |
| Unwashed | 0.50 | -0.42 | 0.44 | -0.47 | -0.80 |
| Water-washed | 0.88 | -0.42 | 0.95 | 0.49 | -0.90 |
| Buffer-washed | -0.88 | -0.21 | 0.25 | 0.86 | -0.43 |

**

**Fig S1** Molecular docking representation of ConA with α-D-mannose. Docking simulations were conducted using CB-Dock2 and SwissDock, while molecular interactions and binding site were examined with PDBsum and visualized through ChimeraX.

*

*

**Fig S2** SEM analyses of strain *S. plymuthica* As3- 5a(5) and cell dimension.

**Fig S3** SEM observation of buffer-washed cells of *S. plymuthica* strain As3-5a(5) after EPS removal with HDMS a) and b) EDS spectra.

**Fig S4** Initial cell density (OD600) versus destained biofilm (OD 595nm) of strains S. plymuthica As3-5a(5). Each data point represents the average reading from three replicates (Mean ± SD, n =3).
